# Successes and challenges of the One Health approach in Kenya over the last decade

**DOI:** 10.1186/s12889-019-6772-7

**Published:** 2019-05-10

**Authors:** Peninah M. Munyua, M. Kariuki Njenga, Eric M. Osoro, Clayton O. Onyango, Austine O. Bitek, Athman Mwatondo, Mathew K. Muturi, Norah Musee, Godfrey Bigogo, Elkanah Otiang, Fredrick Ade, Sara A. Lowther, Robert F. Breiman, John Neatherlin, Joel Montgomery, Marc-Alain Widdowson

**Affiliations:** 1 Division of Global Health Protection, US Centers for Disease Control and Prevention-Kenya, Nairobi, Kenya; 2 Paul G. Allen School for Global Animal Health, Washington State University, Pullman, Washington, USA; 3 Zoonotic Disease Unit, Kenya Ministry of health, Nairobi, Kenya; 4 Zoonotic Disease Unit, Ministry of Agriculture Livestock and Fisheries, Nairobi, Kenya; 5 Center for Global Health Research, Kenya Medical Research Institute, Kisumu, Kenya; 6 Division of Global Health Protection, US Centers for Disease Control and Prevention, Atlanta, GA USA; 7 Emory Global Health Institute, Emory University, Atlanta, GA USA; 8 Division of Global Health Protection, US Centers for Disease Control and Prevention, Dakar, Senegal

**Keywords:** Zoonosis, Cross-sectoral collaboration global health security

## Abstract

More than 75% of emerging infectious diseases are zoonotic in origin and a transdisciplinary, multi-sectoral One Health approach is a key strategy for their effective prevention and control. In 2004, US Centers for Disease Control and Prevention office in Kenya (CDC Kenya) established the Global Disease Detection Division of which one core component was to support, with other partners, the One Health approach to public health science. After catalytic events such as the global expansion of highly pathogenic H5N1 and the 2006 East African multi-country outbreaks of Rift Valley Fever, CDC Kenya supported key Kenya government institutions including the Ministry of Health and the Ministry of Agriculture, Livestock, and Fisheries to establish a framework for multi-sectoral collaboration at national and county level and a coordination office referred to as the Zoonotic Disease Unit (ZDU). The ZDU has provided Kenya with an institutional framework to highlight the public health importance of endemic and epidemic zoonoses including RVF, rabies, brucellosis, Middle East Respiratory Syndrome Coronavirus, anthrax and other emerging issues such as anti-microbial resistance through capacity building programs, surveillance, workforce development, research, coordinated investigation and outbreak response. This has led to improved outbreak response, and generated data (including discovery of new pathogens) that has informed disease control programs to reduce burden of and enhance preparedness for endemic and epidemic zoonotic diseases, thereby enhancing global health security. Since 2014, the Global Health Security Agenda implemented through CDC Kenya and other partners in the country has provided additional impetus to maintain this effort and Kenya’s achievement now serves as a model for other countries in the region.

Significant gaps remain in implementation of the One Health approach at subnational administrative levels; there are sustainability concerns, competing priorities and funding deficiencies.

## Background

More than 75% of emerging and re-emerging infectious diseases are of zoonotic origin including Severe Acute Respiratory Syndrome (SARS), highly pathogenic avian influenza (HPAI), Middle East Respiratory Syndrome (MERS), and Rift Valley Fever (RVF). These can cause explosive global outbreaks resulting in substantial economic and public health burden [1]. Moreover, zoonotic pathogens may spill over to human hosts and in some cases result in a considerable endemic health burden. This is especially true in low resourced and rural settings, where livestock play a central role in daily life and people interact closely with their animals through shared housing and during routine husbandry practices including herding, milking, helping with birthing process and deworming. In addition, various cultural norms in some communities promote consumption of unprocessed livestock products such as unpasteurized milk and uninspected meat. As a result, this expanded and close human-animal interface in countries such as Kenya presents a threat for increased transmission of zoonotic pathogens between animals and humans along the livestock value chain, increasing the likelihood of novel zoonotic pathogens establishing themselves in human populations, the overall endemic burden of common zoonoses, and the threat of infectious disease outbreaks which can threaten global health security.

One Health approach has been defined by the American Veterinary Medical Association as the integrative effort of multiple disciplines working locally, nationally, and globally to attain optimal health for people, animals, and the environment [2]. Globally, the recognition of the threat of emerging and re-emerging zoonoses led to advocacy for the adoption of a One Health approach at country level aimed at strengthening monitoring and response to zoonotic disease risks via a multisectoral, transdisciplinary collaboration [3, 4]. In practice, different countries have approached implementation the principle of One Health variously with varying successes and challenges [5]. In Kenya, creation of a coordination framework between public health and animal (domestic and wildlife) health sector to understand, prevent and control re-emerging and endemic zoonotic diseases has played a central role in the adoption and implementation of the One Health approach.

In Kenya, livestock contributes over 5.5% of the National Gross Domestic Product [6]. About 60% of all cattle, sheep and goats is found in arid and semi-arid areas, where approximately 10 million Kenyans derive their livelihood from livestock. Even in non-arid and semi-arid rural regions of Kenya, most farmers practice mixed farming that includes livestock ownership. For example, among 1500 households participating in a study in Western Kenya, within a non-arid and semi-arid area, 93% of households owned at least one type of livestock with 88% keeping chickens, 55% keeping cattle, 41% keeping goats, and 19% keeping sheep [7].

In 2004, the US CDC, in collaboration with the Kenya Medical Research Institute (KEMRI) and the Kenya Ministry of Health, established the Global Disease Detection Division (GDDD) at CDC Kenya to facilitate building diagnostic and epidemiologic capacity for epidemic prone diseases, conducting public health research of global importance, and contributing to development and use of effective interventions to reduce impact of diseases. Within the GDDD, zoonotic diseases research and a One Health approach were identified as key focus areas with dedicated funding and personnel to lead this effort within the One Health Program formally set up in 2010. The GDDD’s One Health program focused on enhancing collaboration between human and animal health sectors, improving surveillance and diagnostic capacity for the animal health sector and conducting research at the human-animal interface.

Here, we highlight the successes and challenges of the GDDD (now called the Division of Global Health Protection – DGHP) in the last decade, specifically in 1) developing institutional capacity for One Health implementation, 2) strengthening capacity for surveillance and reporting in animal health sector and 3) expanding the research capacity in Kenya and the East Africa region. In addition, we highlight implementation challenges and recommendations to achieve the full benefits of the One Health approach.

### One health institutional capacity

#### A framework for collaboration

Kenya adopted the One Health approach in 2006 by establishing a multi-sectoral committee aligned with global recommendation to coordinate preparedness efforts to prevent the spread HPAI in the wake of the global spread of H5N1. This framework was quickly tested by an outbreak of RVF in the Eastern Africa Region during 2006–2007. Previously, despite the endemicity of RVF and the relative predictability of epidemics, the 1996–1997 RVF outbreak in Kenya caught public health authorities unprepared. Delays in detection and response combined with the lack of local capacity for diagnostic testing (specimens were tested late in the outbreak’s course at the National Institute of Communicable Diseases in South Africa) likely contributed to the large 1996/7 outbreak resulting in 27,500 human infections and 170 deaths in Garissa in North Eastern Kenya. The outbreak was associated with large, but mostly undocumented socio-economic impacts resulting from animal deaths [8, 9]. In contrast, the 2006/07 RVF outbreak in Kenya that was more geographically widespread was characterized by a more timely diagnosis and better coordinated response and resulted in 700 suspected human cases and 90 deaths. This enhanced response could in part be credited to the efforts to build capacity for coordinated outbreak response and communication pathways within the Ministry of Health (MoH) and Ministry of Agriculture, Livestock, and Fisheries (MALF), through increased human capacity, the presence of a multisectoral coordination structure arising from the HPAI preparedness effort and enhanced diagnostic capacity in local government, and improved research infrastructure including a BSL-3 laboratory at KEMRI supported by CDC Kenya [10–12]. The 2006/07 RVF outbreak played a key role in galvanizing collaboration in One Health approaches among government departments, researchers and international organizations to mitigate impacts of future outbreaks and catalyzed the need for clearer understanding and adoption of One Health approach with a focus on supporting animal health surveillance activities [10]. This success with RVF, demonstrated the need to expand and improve the approach for all potentially zoonotic outbreaks, but also to understand, prevent and control endemic zoonotic disease. In Particular a lack of a formal framework for systematic collaboration between government Ministries and among key stakeholders on management of zoonotic disease outbreaks was a critical gap.

**Fig. 1:**
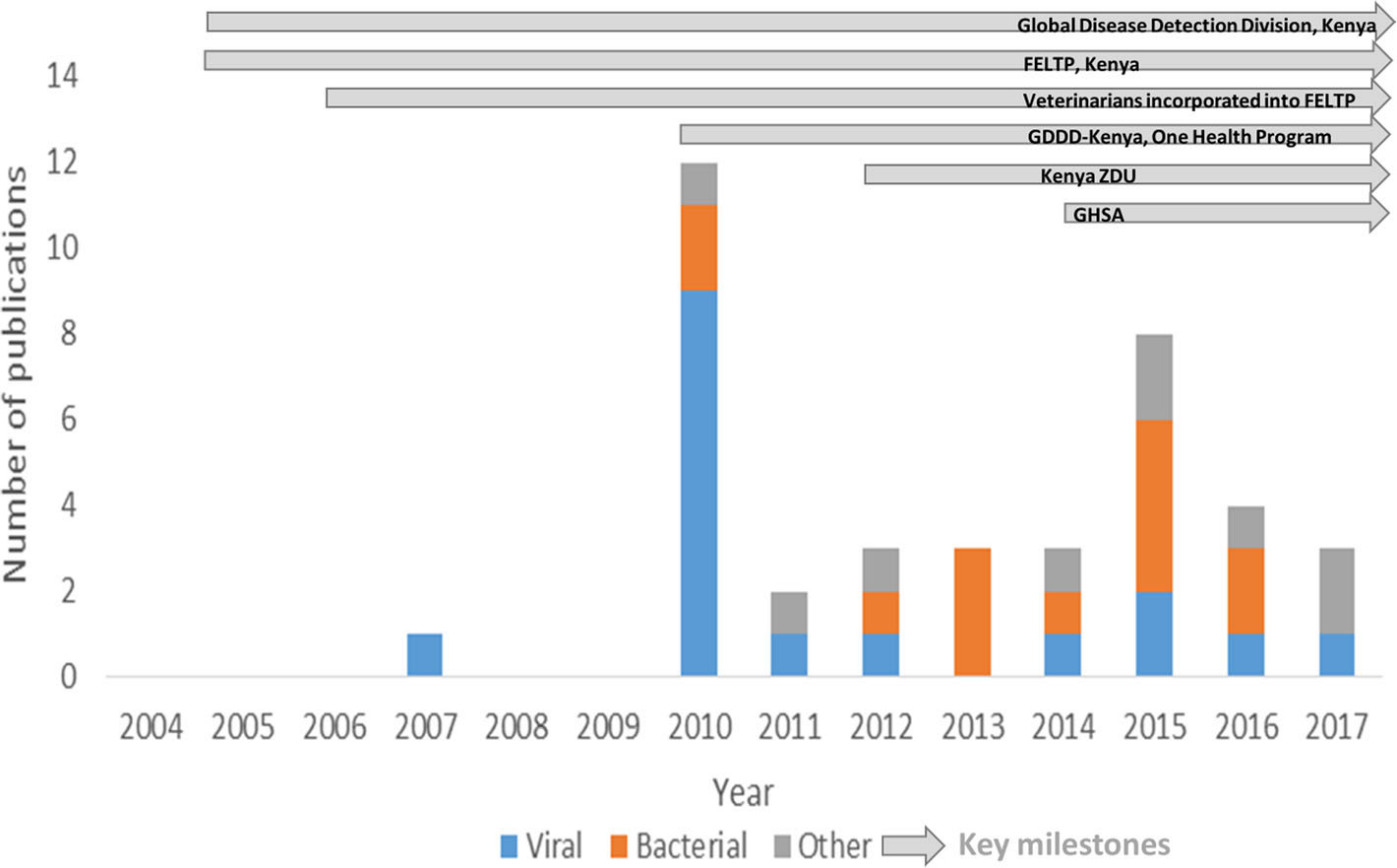
Major milestones on One Health implementation and publications on zoonoses by year, CDC-Kenya, 2004–2017. Global Disease Detection Division (GDDD) now called the Division of Global Health Protection (DGHP); Field Epidemiology and Laboratory Training program (FELTP); Ministry of Health (MOH)

DGHP’s One Health Program worked with other partners including the US Cooperative Biological Engagement Program of the Department of Defense and Biological Engagement Program of the Department of State, to continually advocate for and provide technical and financial assistance to the establishment of a formal collaborative framework between the public and animal health sectors. These efforts led to the formation of a national One Health coordinating office referred to as Zoonotic Disease Unit (ZDU) in 2012 and the process to do this has been laid out previously [13] (Fig. 1).

The ZDU’s key mandate was to act as a focal point of collaboration between the MoH and the MALF with a goal to establish structures and partnerships that promote the One Health approach, to enhance or build zoonotic epidemic and endemic disease surveillance, and to coordinate implementation of control measure and to support public health research in Kenya [13, 14].

In 2013, the governance system in Kenya changed from a centralized to a devolved government of 47 counties; where functions such as public health and animal were undertaken by county governments. This provided an opportunity and need to expand the One Health approach to the subnational (county) level. To cascade the benefits of One Health approach to the county level, the ZDU and its partners have embarked in training and setting up county One Health units supported mainly through CDC Kenya’s Global Health Security Agenda (GHSA) implementing partners. County One Health units focus on initiating or enhancing communication platforms between the health and livestock sectors to improve surveillance and reporting of zoonotic diseases, ensuring rapid joint investigation and response to zoonotic disease outbreaks to mitigate disease impact. As of April 2017, there were 31 of 47 Kenya counties with established county One Health units. Through collaborations with other international partners, there will be county One Health units in all 47 counties by 2019. Sharing of disease outbreak information across sectors and rapid joint outbreak response at county level should help reduce the burden of spillover to humans that acquire zoonotic disease infections as illustrated by an example of an anthrax outbreak in Nakuru County (Table 1).

**Table 1:** Description of an outbreak of anthrax in humans and livestock in Nakuru County, 2016

Anthrax is endemic in Nakuru County, where outbreaks in animals are reported annually. In June 2016, a farmer from one village sent samples from his animal that had died suddenly to the Nakuru Regional Veterinary Investigations Laboratory. These samples tested positive for anthrax. Officers at the regional laboratory notified officers at the sub-county veterinary department, who in turn immediately notified the sub-county department of health in Nakuru County. The sub-county officers of health visited the village to trace the affected farmers’ household, sensitize the community, and health workers and offered prophylaxis treatment to those who had been exposed (eaten meat from the animals or slaughtered/skinned) to dead animals. The veterinary department carried out anthrax ring vaccination of livestock as an outbreak control measure. Upon further records review at the veterinary department, the team identified that a total of 10 cows had died between May and June 2016 of suspected anthrax infection.	
In July 2016, ZDU and Kenya Field Epidemiology and Laboratory Training Program (FELTP) conducted an outbreak investigation at local health centers. A total of 73 exposed people on the investigators’ outbreak line-list were classified as probable cases; 29 of them were traced and 3 (10%) had developed cutaneous anthrax. The majority of those who did not develop clinical disease reported having received prophylaxis treatment at the local health center offered as public health response to the confirmed animal case. The three cutaneous anthrax cases were all exposed from the same animal carcass and all resided in the same village. The timely communication between the health sectors and intervention in those exposed resulted in fewer human cases during this outbreak. The rapid detection and control of this outbreak at its source illustrates the importance of the One Health approach for global health security.	

In 2015, a multidisciplinary team of human, animal and wildlife health experts in surveillance, research and laboratory science drawn from the national and county levels conducted prioritization of zoonotic diseases in Kenya [15]. From a list of 35 zoonotic diseases, the top five were anthrax, trypanosomiasis, rabies, brucellosis and Rift Valley Fever. Based on these findings, development and implementation of disease prevention and control plans for these priority zoonoses with greatest public health impact is being undertaken while promoting collaborative research and surveillance for all the diseases to generate national data and for evaluating control strategies.

#### One Health workforce development

**Fig. 2:**
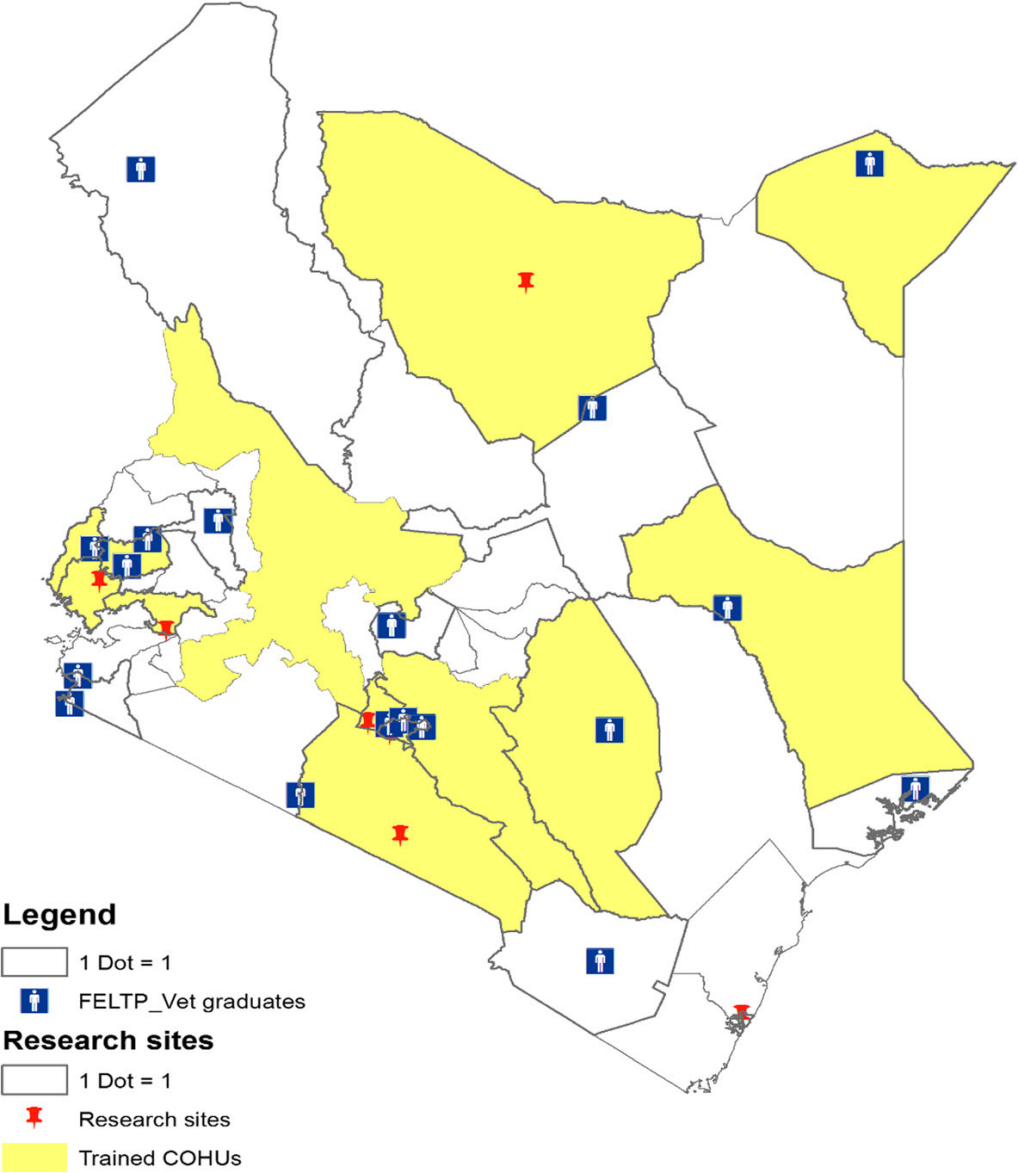
Map of Kenya showing trained County One Health Units, Veterinary Field Epidemiology and Laboratory Training Program (FELTP) graduates and research sites by county. County One Health Units (COHUs)

In 2004, the Kenya Ministry of Health, with support from CDC and other partners, launched Kenya’s Field Epidemiology and Laboratory Training Program (FELTP). This program provides training in applied epidemiology, resulting in a Master’s of Science (MSc), initially offered to medical doctors and laboratory scientists working within government ministries [16]. Since 2006, with increased recognition of the value of One Health, veterinarians have been admitted to the training [16]. By 2017, the Kenya FELTP had 169 medical and 19 veterinary epidemiologists complete the two-year training program. Of these trained veterinarians, all initially returned to government positions, and the majority stayed at national or country level, strengthening collaboration between the human and animal health sector with fellow FELTP graduates participating in joint outbreak response and other activities during their training (Fig. 2).

### Surveillance

Surveillance for animal diseases in most developing countries is designed to detect notifiable and trade sensitive diseases (e.g. foot and mouth disease) and to report to the World Organization for Animal Health (OIE). However, these systems are established to detect animal syndromes and generally without consideration of zoonotic aspects of clusters of animal diseases and the risk that infections may spill-over to humans locally and further afield. This calls for establishment of surveillance platforms that can detect and report cases of these zoonotic diseases in a timely manner, coupled with enhanced collaboration and information sharing between human health and animal health sectors.

In September 2015, the United States’ Pandemic and Forecasting Science Technical Working Group and the Food and Agriculture Organization issued an alert predicting a high likelihood of El Niño-type rainfall and subsequent potential for an RVF epidemic in the Eastern Africa region, and covering the known high risk areas of RVF in Kenya [17, 18]. In response to the alert, CDC Kenya, in collaboration with Kenya MALF (through GHSA funding), set up a mobile phone-based enhanced active surveillance system between November 2015 and February 2016 in 22 (of 47) RVF high-risk counties. The surveillance system collected environmental and livestock monitoring data from resident veterinary officers at county and sub-county level on weekly basis and provided for toll free telephone contact to headquarters in order to report any suspected RVF cases. The veterinary surveillance officers contacted a network of farmers spread out through the sub counties. Over 10,000 reports were submitted with 66 of the syndromic reports meeting the suspect case definition for RVF [19], however none was confirmed, and the conclusion was that no RVF in  livestock had actually occurred. Although an outbreak had not occurred, the potential of one provided an opportunity to test a coordinated, rapid and enhanced surveillance response, highlighting possibility of a more robust and real-time reporting tool in place of a variety of manual and electronic tools that have been used with varied success.

In Kenya, the current surveillance effort is focusing on development and deployment of syndromic surveillance system in domestic and wild animals, using a mobile phone based application that will incorporate reporting and a feedback function to the surveillance officers and has capabilities for routine data analysis and visualization to detect animal disease events of public health concern. The mobile application has adapted manual disease reporting forms currently in use for reporting. Nine syndromes are targeted for reporting: abortion, sudden death, hemorrhagic, neurologic, respiratory, animal bites and oral/foot lesions. Since this mobile phone based application can be downloaded onto any android-based mobile phone device, this reporting tool promises to greatly enhance real-time surveillance within Kenya’s animal health sector. This surveillance is part of institutional capacity building funded by GHSA and targeted to reach up to 10 counties by 2019.

### Zoonotic disease research in Kenya

The burden and transmission dynamics of many zoonotic infections are poorly understood in developing countries, including Kenya, which can challenge the progress of disease control programs to reduce burden and impact. In 2005, KEMRI and the DGHP in Kenya established the Population Based Infectious Disease Surveillance (PBIDS) platforms in a rural site in Western Kenya and an urban site in Nairobi to define the burden, etiologies and risk factors of common infectious disease syndromes (fever, jaundice, diarrhea and respiratory illness) among others [20, 21]. Additionally, in 2008 through collaborative partnerships with the Wellcome Trust, zoonotic disease research was started among farmers and animals within PBIDS. The zoonotic disease research in DGHP has catalyzed additional studies within the PBIDS platform with other partners such as Washington State University and developed further platforms in different sites in the country with the overall focus of generating disease data to inform public health actions (Fig. 1). Some examples of these are outlined below.

#### Q-fever

*Coxiella burnetti* is on the US Federal Select Agent list and was first reported in Kenya over half a century ago, but since the late 1970’s there had been no further study of this pathogen. In 2009, KEMRI and CDC Kenya carried out retrospective studies on archived human sera collected between 2007 and 2008 in the PBIDS platform and cross-sectional studies carried out in the same site in 2009 among cattle, sheep and goats and the vector ticks to determine the sero-prevalence of the disease [22]. These pivotal studies by CDC and KEMRI triggered a sustained interest among research groups who showed variable but high sero-prevalence in humans and the reservoir domestic animals in different eco-systems in Kenya and highlighted *Coxiella burnetti* as a key etiology for consideration for undifferentiated fever in communities keeping livestock [23, 24]. In 2015, data from these studies were used during a One Health Zoonotic Disease Prioritization workshop in Kenya, where Q-fever was identified as one of the diseases that would benefit from integrated prevention and control programs using the One Health approach [15].

#### Brucellosis

CDC Kenya and the ZDU implemented a study in three counties among humans and their livestock and found a varying (2.4–46.4%) seroprevalence in humans and 1.2–13.5% in livestock among the counties, largely associated with cultural practices around livestock and their products particularly unpasteurized milk and low knowledge levels on brucellosis. This underscored the need for targeted public health messaging, effective diagnostic capacity in local hospitals and systematic control programs for brucellosis in animals [25].

#### Rift Valley fever

The momentum built around the 2006–2007 RVF outbreak in Kenya progressed to robust research projects by multisectoral collaborative research groups. A key output from learning the lessons on preparedness from the 2006–2007 outbreak is the RVF decision support tool kit for Chief Veterinary officers in the Horn of Africa region to support evidence-based actions to mitigate the impact of RVF outbreaks when they occur [26]. This tool has been adapted into the RVF integrated preparedness and response plan for Kenya and was applied in late 2015 when RVF was predicted in Kenya and the Eastern Africa region as described previously.

Other DGHP- Kenya research work on RVF described climatic, geographic, and geologic predictive factors associated with occurrence of RVF in Kenya [27, 28]. These data taken together with a historical review of RVF outbreaks since 1912, were used to generate a risk map for RVF in Kenya [27, 29, 30]. A temporal spatial mapping of the RVF outbreaks in the Eastern Africa region followed by molecular analysis of viruses isolated from humans, animals and mosquitoes found foci-specific viral lineages suggesting de novo activation of viruses in specific outbreak sites rather than spatial spread from the initial outbreak site to another [31]. This knowledge was useful in defining the endemic nature of RVF in certain regions in Kenya and subsequent studies on factors associated with endemicity, outbreak flare-ups and factors associated with human morbidity and mortality [27, 32]. To address safety concerns surrounding the locally available RVF vaccine, a field trial to evaluate the safety and efficacy of new RVF clone 13 vaccine was conducted in Kenya in collaboration with Kenya Ministry of Agriculture Livestock and Fisheries [33–35]. The results showed the vaccine as a promising tool, being safe and with high immunogenicity in sheep and goats and moderate immunogenicity in cattle under field conditions. This vaccine has been earmarked for registration locally.

#### Rickettsia

Between 2006 and 2008 a hospital based study conducted by KEMRI and CDC Kenya in North Eastern Kenya identified for the first time, *Rickettsia felis*—an agent of flea-borne spotted fever in 3.7% of patients [36]. A second study (2007–2010) in the PBIDS Lwak health facility in Western Kenya reported 57% seroprevalence of antibodies versus Rickettsial species in archived human sera. Rickettsia were detected by PCR in 7.2% of febrile patients, which was 2-fold higher than in the afebrile patients suggesting that Rickettsial pathogens are an important differential diagnosis among patients with undifferentiated fever in this region [37]. Subsequently cross-sectional studies in PBIDS platforms found previous exposure to spotted fever group rickettsia in goats (43%), sheep (23%) and cattle (1%) and in majority (> 90%) of *Amblyomma variegatum* tick pools and in 66% of flea pools [38]. Further these studies identified, isolated and sequenced a novel (to the world) rickettsial pathogen, *Rickettsia asemboensis* in cat and dog fleas in western Kenya.

#### Middle East respiratory syndrome coronavirus

High seroprevalence of Middle East Respiratory Syndrome Corona Virus (MERS COV) among camels has been reported in Kenya and other countries in Africa [39, 40]. Kenya has over three million camels reared in the arid northern part of the country. CDC Kenya in collaboration with KEMRI assessed whether persons exposed to seropositive camels (90% sero-prevalence) at household level had serological evidence of infection. None of the 760 persons tested with well-characterized exposure to camels and camel products (milk) showed evidence of previous exposure MERS COV [41]. To test the hypothesis that perhaps a different strain of MERS COV that is less transmissible from camels to humans is circulating in camels in Kenya we have set up a study to detect and isolate MERS virus strain circulating in camels in Kenya for comparison with strains circulating elsewhere and addition expanded an enhanced surveillance of respiratory illness to Marsabit where camel pastoralists reside to detect any cases in humans.

#### Potential zoonotic pathogens in small mammals

In 2008, KEMRI and CDC conducted a study in the urban (Nairobi) Kibera PBIDS platform with rodents captured in and around houses and reported a diversity of pathogenic leptospires [42]. Two studies carried out by KEMRI and CDC on *Bartonella* in rodents and bats captured in or around homesteads in Kibera PBIDS platform found *Bartonella* strains that are closely related to known human *Bartonella* species [43]. In addition, from a variety of bats captured in multiple sites across Kenya, a high prevalence and diversity of *Bartonella* species were identified [44]. Detection of these pathogens in these animal reservoirs suggests potential for exposure and transmission to humans in different settings. Future work is underway to understand the contribution of these pathogens as etiologies for undifferentiated fever in humans using multi-pathogen detection assay such as the AFI TaqMan array card in several sites including the PBIDs platform in Western Kenya.

#### Linking animal ownership and health status to human health outcomes

Since 2013, CDC and KEMRI in collaboration with Washington State University have conducted animal (cattle, sheep, goats, and poultry) syndromic surveillance in 1500 households in Western Kenya, a subset of the on-going PBIDS platform [7]. This integrated and unique study design allows for measuring of the impact of livestock diseases on human health and socio-economic status at household level. Preliminary data analysis reported at household level, showed strong association between cumulative human and animal illness though the mechanism for this association was not clear [7]. Further work looking at  the linkage of human health and owning livestock through the nutritional pathway as well as assessing the level of microbiome development among children at household level has yielded insights into the impact of animal ownership and animal source foods on growth and development of children under 5 years old as well as the factors associated with level of microbiome sharing [45, 46]. There are on-going studies on impact of interventions to reduce diseases in animals on health and socio-economic status of households.

In summary, the CDC, KEMRI and collaborators investments in research on zoonotic diseases has generated credible and useful data on occurrence, identified new pathogens and etiologies of common syndromes, clarified the ecology of disease occurrence and overall contributed in the formulation of science-based interventions in endemic zoonotic diseases. In addition, the innovative and unique linked human-animal interface study designs have highlighted the benefits of adopting a One Health approach in research to study zoonoses and complex interlinked human and animal health relationships. Overall, 30 publications covering viral, bacterial and other (mainly reviews and topics on disease ecology) have been realized from this work (Fig. 1).

### Challenges

While zoonotic diseases spill-over to human populations and the concept of controlling infections at source is well appreciated, the animal health sector continues to be under-resourced in critical elements of surveillance and reporting of animal diseases and laboratory diagnosis. In addition, setting up surveillance programs is resource intensive with few partners providing support. Convincing policy makers of the benefit of planning and investing in animal surveillance for public health gain is often challenged where data on burden of zoonoses are scanty and when the threat is not immediately apparent zoonotic and/or not an existing emerging disease threat. On a positive note, in 2013 DGHP Kenya funded the development of a rabies elimination plan for Kenya [14] that has since attracted funding from various MALF and MOH development partners for rabies elimination activities including mass dog vaccination, enhanced rabies surveillance in humans and animals, enhanced management of dog bite cases and operational research. Finally, the Kenya ZDU has not fully incorporated the environment health sector to be fully compliant with the scope of One Health approach. This has in part been due to lack of clearly designated government ministry that represents the environment sector. Currently efforts have been made to include an ecologist as a core personnel to the ZDU to provide environmental health expertise. In addition, the strategic plan for the implementation of One Health is being reviewed to reflect the progress made and identify strategies for institutionalizing One Health at sub-national level.

The change of Kenya’s governance structure from a central to a devolved system in 2012 necessitated a different approach in the implementation of One Health. The extent of adoption of the One Health approach has been remarkable at the national level. However, at the sub-national (County and sub-county levels) where most disease management decisions are made, more progress is needed. With GHSA support, the ZDU has made concerted efforts to establish functional One Health units at county levels to enhance coordination and communication of key ministries and stakeholders for surveillance and disease outbreak response; however the benefits of these efforts are currently being evaluated. Finally, sustainability of the current progress and efforts are not guaranteed due to the reliance on donor funding to implement these One Health activities. This is a challenge that goes beyond One Health implementation and is largely appreciated for many donor-initiated efforts. Most governments have a host of competing interests including constant outbreaks of epidemic prone diseases such as cholera, other infectious diseases and non-communicable diseases within the health sector and focusing on promotion of international trade in animals and animal products that often make the importance of zoonotic diseases pale in comparison. Commendably, ZDU has initiated advocacy plans to lobby for government support through the line ministries to maintain One Health activities beyond the current GHSA initiative.

## Conclusions

Since 2006, CDC Kenya has successfully supported and collaborated with Kenyan government institutions to establish a sustainable One Health program at national and county levels; a process catalyzed by emerging zoonotic threats such as RVF and H5N1. The results have been the establishment of an effective cross-sectoral coordinating government unit (ZDU), an enhanced surveillance system in domestic and wild animals that meets the needs of animal and human health, a workforce trained in the One Health approach, improved outbreak investigations and a robust and productive public health scientific program including the discovery of zoonotic pathogens new to the world. The adoption of the One Health program and approach in Kenya has led to rapid detection and control of zoonotic disease outbreaks at their source and thereby enhanced global health security. These achievements have allowed for advocacy and informed decisions to be made on the control and prevention of zoonotic pathogens and have identified gaps in diagnosis and surveillance. However, challenges remain in sustainability, veterinary laboratory diagnosis and resources to implement more comprehensive control and prevention measures.

Since zoonotic infections continue to  impose a health burden on population, and new zoonoses can emerge in any country and spread globally, the regional and global adoption of the One Health agenda is a key capacity of the global health security agenda. Lessons learnt from Counties in Kenya are applicable to establish One Health programs throughout Kenya, the African region, and beyond.

## Abbreviations

CDC: Centers for Disease Control and Prevention
DGHP: Division of Global Health Protection
GDDD: Global Disease Detection Division
HPAI: Highly pathogenic avian influenza
KEMRI: Kenya Medical Research Institute
MERS COV: Middle East Respiratory Syndrome Corona Virus
RVF: Rift Valley Fever
ZDU: Zoonotic Disease Unit
